# Antiplatelet Therapy Strategies in Patients with High Bleeding Risk Treated with Complex Percutaneous Coronary Intervention: A Descriptive Study

**DOI:** 10.1016/j.cjco.2026.06.009

**Published:** 2026-07-01

**Authors:** Éloi Ravet-Claret, Susan Chen, Adèle Goulet, Dan Tcaciuc, Jamey Scraire, Léa Turgeon-Mallette, Marie Lordkipanidzé, Marie-France Gauthier, Julien Quang Le Van

**Affiliations:** a Faculty of Pharmacy, Université de Montréal, Montreal, Quebec, Canada; b Montreal Heart Institute, Montreal, Quebec, Canada

**Keywords:** dual antiplatelet therapy, bleeding risk, complex percutaneous coronary intervention, acute coronary syndrome

## Abstract

**Background:**

Following acute coronary syndrome (ACS) treated by percutaneous coronary intervention (PCI), guideline-directed dual antiplatelet therapy (DAPT) typically consists of 12 months of aspirin plus a P2Y_12_ inhibitor. Recent trials support antiplatelet de-escalation strategies in patients at high bleeding risk (HBR), whereas patients undergoing complex PCI are at increased thrombotic risk and may benefit from extended DAPT. The overlap between these groups with high bleeding risk and high thrombotic risk in contemporary practice is not well characterized.

**Methods:**

Patients undergoing PCI for ACS were randomly sampled from those hospitalized at the Montreal Heart Institute between May 1, 2024 and April 30, 2025. Data were extracted from electronic health records. HBR was assessed using the **Pre**dicting Bleeding **C**omplications in Patients Undergoing Stent **I**mplantation and **S**ubsequ**e**nt Dual Antiplatelet Therapy-**H**igh **B**leeding **R**isk (PRECISE-HBR) score, and complex PCI was assessed using guideline-based criteria. The primary objective was to estimate the proportion of patients meeting both criteria; secondary objectives described discharge antiplatelet strategies across risk groups.

**Results:**

Of the 309 participants sampled, 35% were identified as being at HBR, 43% underwent complex PCI, and 17.5% (95% confidence interval, 13.3%-21.7%) met both HBR and complex PCI criteria. De-escalation strategies were infrequently prescribed across risk groups, even among patients at HBR without complex PCI. Among those not receiving long-term anticoagulation, only 4.2% received a de-escalation strategy.

**Conclusions:**

Nearly 1 in 6 patients undergoing PCI for ACS met both HBR and complex PCI criteria. In this setting, clinicians appeared to favour standard-duration clopidogrel-based DAPT rather than de-escalation approaches. These findings underscore the need for practical guidance to support individualized antiplatelet decision-making in patients with competing bleeding and ischemic risks.

## Central Illustration

**Figure:**
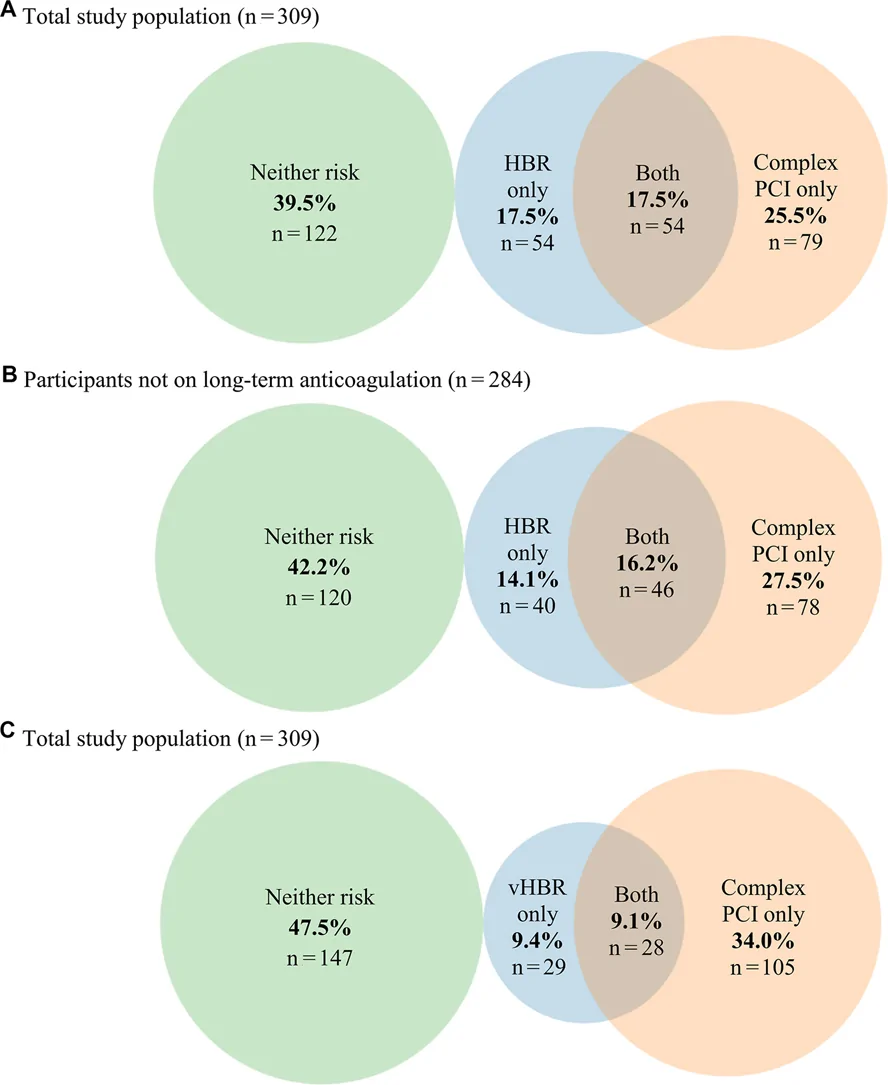

## Résumé

**Contexte:**

À la suite d’un syndrome coronarien aigu (SCA) traité par intervention coronarienne percutanée (ICP), la bithérapie antiplaquettaire (BTAP) recommandée par les lignes directrices consiste généralement en un traitement de 12 mois associant l’aspirine à un inhibiteur de P2Y_12_. Des essais récents soutiennent les stratégies de désescalade du traitement antiplaquettaire chez les patients présentant un risque hémorragique élevé (RHE), tandis que ceux ayant subi une ICP complexe sont exposés à un risque thrombotique accru et pourraient bénéficier d’une prolongation de la BTAP. Le chevauchement entre ces groupes à haut risque hémorragique et à haut risque thrombotique dans la pratique clinique actuelle n’est pas bien caractérisé.

**Méthodologie:**

Des patients traités par ICP pour un SCA ont été sélectionnés de manière aléatoire parmi ceux hospitalisés à l’Institut de cardiologie de Montréal entre le 1^er^ mai 2024 et le 30 avril 2025. Les données ont été extraites des dossiers médicaux électroniques. Le RHE a été évalué selon le score PRECISE-HBR, tandis que la complexité de l'ICP a été déterminée selon des critères issus des lignes directrices. L’objectif principal était d’estimer la proportion de patients répondant à ces deux critères; les objectifs secondaires consistaient à décrire les stratégies antiplaquettaires à la sortie de l’hôpital selon les groupes de risque.

**Résultats:**

Sur les 309 participants de l’échantillon, 35 % ont été identifiés comme présentant un RHE, 43 % ont bénéficié d’une ICP complexe et 17,5 % (intervalle de confiance à 95 %, 13,3-21,7) répondaient à la fois aux critères de RHE et d’ICP complexe. Les stratégies de désescalade ont été rarement prescrites dans l’ensemble des groupes de risque, même chez les patients présentant un RHE sans ICP complexe. Parmi ceux ne recevant pas d’anticoagulation à long terme, seuls 4,2 % ont bénéficié d’une stratégie de désescalade.

**Conclusions:**

Près d’un patient sur six bénéficiant d’une ICP pour un SCA répondait à la fois aux critères de RHE et d'ICP complexe. Dans ce contexte, les cliniciens semblaient privilégier une BTAP à base de clopidogrel de durée standard plutôt que des approches de désescalade. Ces résultats soulignent la nécessité de formuler des recommandations pratiques afin de soutenir une prise de décision individualisée concernant le traitement antiplaquettaire chez les patients présentant à la fois un risque hémorragique et un risque ischémique.

Following an acute coronary syndrome (ACS) treated with percutaneous coronary intervention (PCI), dual antiplatelet therapy (DAPT) is recommended for 12 months, typically combining aspirin with a potent P2Y_12_ inhibitor (ticagrelor or prasugrel).^1^, ^2^, ^3^ Contemporary evidence supports de-escalation strategies to reduce bleeding risk in selected patients, particularly those at high bleeding risk (HBR). These strategies include shortening DAPT after ≥ 1 month followed by single antiplatelet therapy, or switching after ≥ 1 month from potent DAPT to a clopidogrel-based regimen.^4^, ^5^, ^6^, ^7^ Conversely, patients undergoing complex PCI are at increased thrombotic risk and may derive benefit from more intensive and prolonged antiplatelet therapy.^8^^,^^9^

This context creates a therapeutic challenge when patients present with both high bleeding and high thrombotic risk features. Contemporary cardiology guidelines provide recommendations for HBR and complex PCI individually, but they do not offer specific guidance for patients who meet both criteria.^1^, ^2^, ^3^ In addition, to our knowledge, only one randomized trial comparing a de-escalation strategy to standard-duration DAPT included patients with both HBR and complex PCI features.^4^ Although DAPT de-escalation was associated with similar ischemic outcomes regardless of PCI complexity, patients meeting both HBR and complex PCI criteria represented only 26% of the trial population.^10^ Therefore, evidence specific to this subgroup remains limited, and the analysis was likely underpowered to guide treatment decisions. Although HBR and complex PCI have been described individually in prior studies, the prevalence of their coexistence in contemporary PCI practice remains poorly characterized.^11^^,^^12^ Together, these gaps highlight the need for contemporary real-world data to better define the prevalence and management of this subgroup with overlapping high risk.

The primary objective of this study was to estimate the proportion of patients undergoing PCI who met both HBR and complex PCI criteria. Secondary objectives were to describe discharge antiplatelet strategies across all combinations of HBR and complex PCI status.

## Material and Methods

### Participant selection

This descriptive observational study included patients admitted to the coronary care unit of the Montreal Heart Institute, a tertiary cardiology hospital in Canada, between May 1, 2024 and April 30, 2025. The 1-year study period began 6 months after publication of the 2023 Canadian Cardiovascular Society/Canadian Association of Interventional Cardiology (CCS/CAIC) antiplatelet guidelines to allow time for practice adoption and to evaluate the uptake of newer antiplatelet strategies.^1^ Eligible patients were adults aged 18 years or older hospitalized for ACS who underwent PCI with stent implantation. Patients were excluded if they underwent cardiac surgery during the index hospitalization, as perioperative bleeding risk strongly influences antiplatelet selection. We also excluded those with prior stent thrombosis, antiphospholipid syndrome, or thrombophilia, for whom de-escalation strategies may be inappropriate due to very high thrombotic risk.

The study protocol (#2025-3499) was approved by the Montreal Heart Institute Research Ethics Board, which waived the requirement for individual patient consent.

### Data collection

Data were collected from electronic health records, which provided access to cardiac catheterization reports, laboratory test results, medical notes, and discharge prescriptions. Demographic variables included age, sex, weight, height, and body mass index. HBR was assessed using the **Pre**dicting Bleeding **C**omplications in Patients Undergoing Stent **I**mplantation and **S**ubsequ**e**nt Dual Antiplatelet Therapy-**H**igh **B**leeding **R**isk (PRECISE-HBR) score, a validated risk prediction tool derived and externally validated in large PCI cohorts.^4^^,^^13^, ^14^, ^15^ This score estimates the probability of major bleeding occurring within 1 year after PCI and identifies patients at high (> 4%) or very high (> 6%) bleeding risk (vHBR). We selected PRECISE-HBR as the primary definition for all HBR and vHBR analyses because it provides an individualized absolute risk estimate, allows further stratification of bleeding risk severity, and has demonstrated improved discrimination compared with earlier tools, including the **Pre**dicting Bleeding **C**omplications in Patients Undergoing Stent **I**mplantation and **S**ubsequ**e**nt **D**ual **A**nti**p**latelet **T**herapy (PRECISE-DAPT) score and the Academic Research Consortium for High Bleeding Risk (ARC-HBR) criteria.^16^^,^^17^ In contrast, the ARC-HBR framework classifies patients dichotomously as being at HBR or non-HBR, defined as the presence of at least 1 major criterion or 2 minor criteria, corresponding to an expected major bleeding risk ≥ 4% at 1 year.^17^ To calculate the PRECISE-HBR score for each patient, the following variables were extracted: estimated glomerular filtration rate; serum hemoglobin and serum white blood cell count at baseline; history of spontaneous bleeding requiring medical attention; concomitant use of oral anticoagulants; presence of platelet count < 100 × 10^9^/L; diagnosis of cirrhosis with portal hypertension; active malignancy within the past 12 months (excluding non-melanoma skin cancers); chronic bleeding diathesis; major surgery or trauma within 30 days before PCI; and chronic use of nonsteroidal anti-inflammatory drugs or corticosteroids (planned daily intake ≥ 4 d/wk). Because all PRECISE-HBR variables, except for white blood cell count, are also included in the ARC-HBR framework, these variables were also used for an exploratory sensitivity analysis based on available ARC-HBR criteria. However, selected ARC-HBR-specific criteria were not formally collected, including prior spontaneous intracranial hemorrhage, traumatic intracranial hemorrhage within 12 months, brain arteriovenous malformation, moderate or severe ischemic stroke within 6 months, nondeferrable major surgery while receiving DAPT, and prior ischemic stroke that does not meet a major ARC-HBR criterion.

To assess procedural complexity and identify patients at higher thrombotic risk, we applied the complex PCI criteria outlined in the 2023 CCS/CAIC guidelines.^1^ The following procedural variables were collected: total stent length ≥ 60 mm, bifurcation with 2 stents; stenting in the left main coronary artery; implantation of ≥ 3 stents; treatment of ≥ 3 vessels or ≥ 3 lesions; use of atherectomy; intervention for chronic total occlusion; and intervention on a bypass graft. Patients were classified as having undergone a complex PCI if at least one of these criteria was present. Additional variables included antiplatelet therapy prescribed at discharge, the intended duration of DAPT in patients without long-term anticoagulation, the type of ACS index event (ST-segment elevation myocardial infarction [STEMI], non-ST-segment elevation myocardial infarction [NSTEMI], unstable angina), presence of diabetes, and history of complex PCI within 12 months prior to the current hospitalization.

### Statistical analysis

For the primary objective, a total sample size of 309 patients was calculated to estimate the proportion of patients meeting both HBR and complex PCI criteria with a 2-sided 95% confidence interval (CI) with a margin of error of 3.1%, assuming an expected prevalence of 8.6%. To ensure temporal representativeness, patients were sampled in 7-day blocks throughout the study period, and within each block, an equal number of patients were randomly selected until the target sample size was reached. Continuous variables were summarized as medians with interquartile range (IQR), and categorial variables as counts and percentages.

We estimated proportions with 95% CIs using the Wald method for 4 mutually exclusive combinations of PRECISE-HBR-defined HBR and complex PCI (HBR only, complex PCI only, both HBR and complex PCI, and neither HBR nor complex PCI), in the overall cohort and among participants not on long-term anticoagulation. Analyses were repeated using PRECISE-HBR-defined vHBR instead of HBR. Antiplatelet strategy use was described within these subgroups among patients not on long-term anticoagulation. In an exploratory sensitivity analysis, only the primary analysis was repeated using HBR defined according to available ARC-HBR criteria instead of PRECISE-HBR. For each subgroup, we reported the proportion of patients treated with the following: (i) standard-duration DAPT (12 months) with clopidogrel; (ii) standard-duration potent DAPT (12 months with ticagrelor or prasugrel); (iii) short-duration DAPT defined as 1-3 months of DAPT followed by single antiplatelet therapy; and (iv) reduced-intensity DAPT, defined as switching after 1-3 months from ticagrelor- or prasugrel-based DAPT to clopidogrel-based DAPT. In a post-hoc analysis, we evaluated whether predicted 1-year major bleeding risk differed across increasing levels of PCI complexity (defined by the number of complex PCI criteria).

## Results

Of the 349 patients screened, 309 were included in the study (Supplemental Fig. S1). The main reasons for exclusion were PCI performed for indications other than ACS (n = 15), planned cardiac surgery during the index hospitalization (n = 10), and history of stent thrombosis (n = 10). The median age was 69 years (IQR 61-77) and 84 patients (27.2%) were women (Table 1). The index ACS event was STEMI in 138 patients (44.7%), NSTEMI in 90 patients (29.1%) and unstable angina in 81 patients (26.2%). Missing data were limited to 6 patients with unknown weight and 18 with unknown height.

**Table 1:** Baseline characteristics of study population

Characteristics	Total population (n = 309)
**Demographics**	
Age, Y	69 (61–77)
Sex, female	84 (27.2)
Body mass index, kg/m^2^	27.0 (24.8–30.9)
**Index ACS event**	
STEMI	138 (44.7)
NSTEMI	90 (29.1)
Unstable angina	81 (26.2)
**Admission laboratory values**	
Hemoglobin, g/L	142 (129–153)
eGFR, mL/min per 1.73 m^2^	79.8 (67.6–93.1)
WBC, count x 10^9^/L	8.0 (6.5–9.7)
**Medical HISTORY**	
Type II diabetes	89 (28.8)
Previous bleeding	3 (1.0)
Long-term oral anticoagulation	25 (8.1)
Platelets < 100 x 10^9^/L	6 (1.9)
Hemorrhagic diathesis	0 (0)
Portal hypertension cirrhosis	2 (0.6)
Active neoplasia in past 12 months	13 (4.2)
Recent trauma or major surgery	2 (0.6)
NSAID or corticosteroid use	7 (2.3)
Clinical risk features	
**1-Y bleeding risk, %**	3.1 (2.4–4.8)
**Complex PCI criteria number**	133 (43.0)
1	43 (13.9)
2	26 (8.4)
3	38 (12.3)
≥ 4	26 (8.4)

Data are expressed as n (%) or median (interquartile range).

ACS, acute coronary syndrome; eGFR, estimated glomerular filtration rate; IQR, interquartile range; NSAID, nonsteroidal anti-inflammatory drugs; NSTEMI, non-ST-segment elevation myocardial infarction; PCI, percutaneous coronary intervention; STEMI, ST-segment elevation myocardial infarction; WBC, white blood cell count.

Overall, 35% of patients met the criteria for HBR according to the PRECISE-HBR score, 43% underwent complex PCI, and 17.5% (95% CI, 13.3%-21.7%) met both HBR and complex PCI criteria (Central Illustration; Supplemental Table S1**)**. Among the 284 patients who were not on long-term anticoagulation, 16.2% (95% CI, 11.9%-20.5%) met both HBR and complex PCI criteria. Using the more restrictive vHBR definition, 9.1% (95% CI, 5.9%-12.3%) met both vHBR and complex PCI criteria. In the exploratory sensitivity analysis using available ARC-HBR criteria, 28.8% of patients met HBR criteria, including 57 patients who met at least 1 major criterion and 32 patients who met at least 2 minor criteria, and 13.9% (95% CI, 10.1%-17.8%) met both HBR and complex PCI criteria (Supplemental Fig. S2; Supplemental Table S2). The distribution of PRECISE-HBR components across bleeding-risk strata is shown in Table 2. In HBR patients, the median predicted 1-year major bleeding risk was 6.1% (IQR 4.6%-8.9%), increasing to 8.9% (IQR 6.7%-11.3%) in those classified as vHBR, representing 53% of the HBR group. Compared with non-HBR patients, those classified as HBR were older (aged 79.5 vs 64 years), had lower estimated glomerular filtration rate (67.8 vs 85.7 mL/min per 1.73 m^2^), had lower hemoglobin levels (125 vs 149 g/L), and were more frequently receiving long-term anticoagulation (20.4% vs 1.5%). Similar associations were observed in the vHBR subgroup. Overall, 176 patients did not meet complex PCI criteria, whereas 43 met 1 criterion, 26 met 2, 38 met 3, and 26 met 4 or more criteria (Fig. 1; Supplemental Table S3). Median predicted 1-year major bleeding risk ranged from 2.9% to 4.0% across these groups, defined by increasing numbers of complex PCI criteria. Among patients meeting complex PCI criteria, the most frequent components were treatment of ≥ 3 lesions (63.2%), total stent length ≥ 60 mm (61.7%), and implantation of ≥ 3 stents (53.4%; Fig. 2).

**Central Illustration:**
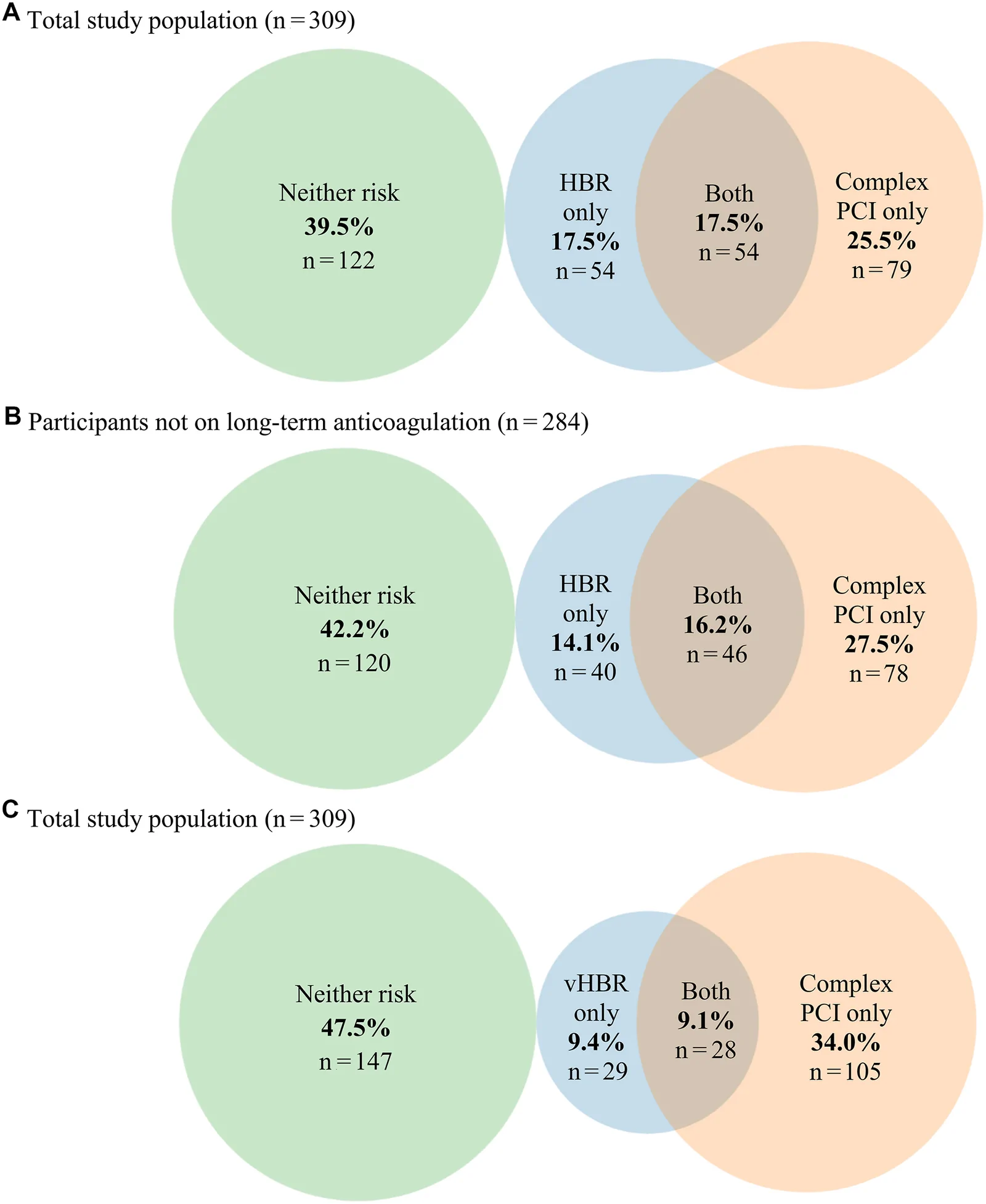
Proportional overlap between high bleeding risk (**Pre**dicting Bleeding **C**omplications **i**n Patients Undergoing **S**tent Implantation and Subs**e**quent **D**ual **A**nti **P**latelet **T**herapy-high bleeding risk [PRECISE-HBR]) and complex percutaneous coronary intervention (PCI). Three Venn diagrams illustrate the overlap between high bleeding risk (HBR [ ≥ 4%]) and complex PCI in the study population. **Circle areas** are proportional to the number of patients in each category. (**A**) overall cohort, using an HBR definition of PRECISE-HBR ≥ 4%. (**B**) The same analysis restricted to patients not receiving long-term oral anticoagulation. (**C**) The overall cohort using a very high bleeding risk (vHBR [≥ 6%]) definition of PRECISE-HBR ≥ 6%.

**Table 2:** Distribution of **Pre**dicting Bleeding **C**omplications **i**n Patients Undergoing **S**tent Implantation and Subs**e**quent **D**ual **A**nti **P**latelet **T**herapy-high bleeding risk (PRECISE-HBR) score criteria by patient bleeding risk stratification

PRECISE-HBR criteria	Total population (n = 309)	Not HBR (n = 201)	HBR (≥ 4%) (n = 108)	vHBR (≥ 6%) (n = 57)
Age, Y	69 (61–77)	64 (57–71)	79.5 (73.0–83.3)	81 (75–87)
Hemoglobin, g/L	142 (129–153)	149 (140–158)	125 (118–132)	121 (114–125)
eGFR, mL/min per 1.73 m^2^	79.8 (67.6–93.1)	85.7 (74.9–97.2)	67.8 (53.0–80.1)	58 (42–70.5)
WBC, count x 10^9^/L	8.0 (6.5–9.7)	8.0 (6.5–9.7)	8.1 (6.4–10.2)	8.3 (6.7–10.3)
Previous bleeding	3 (1.0)	0 (0)	3 (2.8)	2 (3.5)
Long-term oral anticoagulation	25 (8.1)	3 (1.5)	22 (20.4)	17 (29.8)
Platelets < 100 x 10^9^/L	6 (1.9)	1 (0.5)	5 (4.6)	2 (3.5)
Hemorrhagic diathesis	0 (0)	0 (0)	0 (0)	0 (0)
Portal hypertension cirrhosis	2 (0.6)	1 (0.5)	1 (0.9)	1 (1.8)
Active neoplasia in past 12 months	13 (4.2)	5 (2.5)	8 (7.4)	5 (8.8)
Recent trauma or major surgery	2 (0.6)	1 (0.5)	1 (0.9)	1 (1.8)
NSAID or corticosteroid use	7 (2.3)	3 (1.5)	4 (3.7)	4 (7)
1-Y bleeding risk (%)	3.1 (2.4–4.8)	2.6 (1.9–3.1)	6.1 (4.6–8.9)	8.9 (6.7–11.3)

Data are expressed as n (%) or median (interquartile range).

eGFR, estimated glomerular filtration rate; HBR, high bleeding risk; NSAID, nonsteroidal anti-inflammatory drugs; vHBR, very high bleeding risk; WBC, white blood cell count.

**Figure 1:**
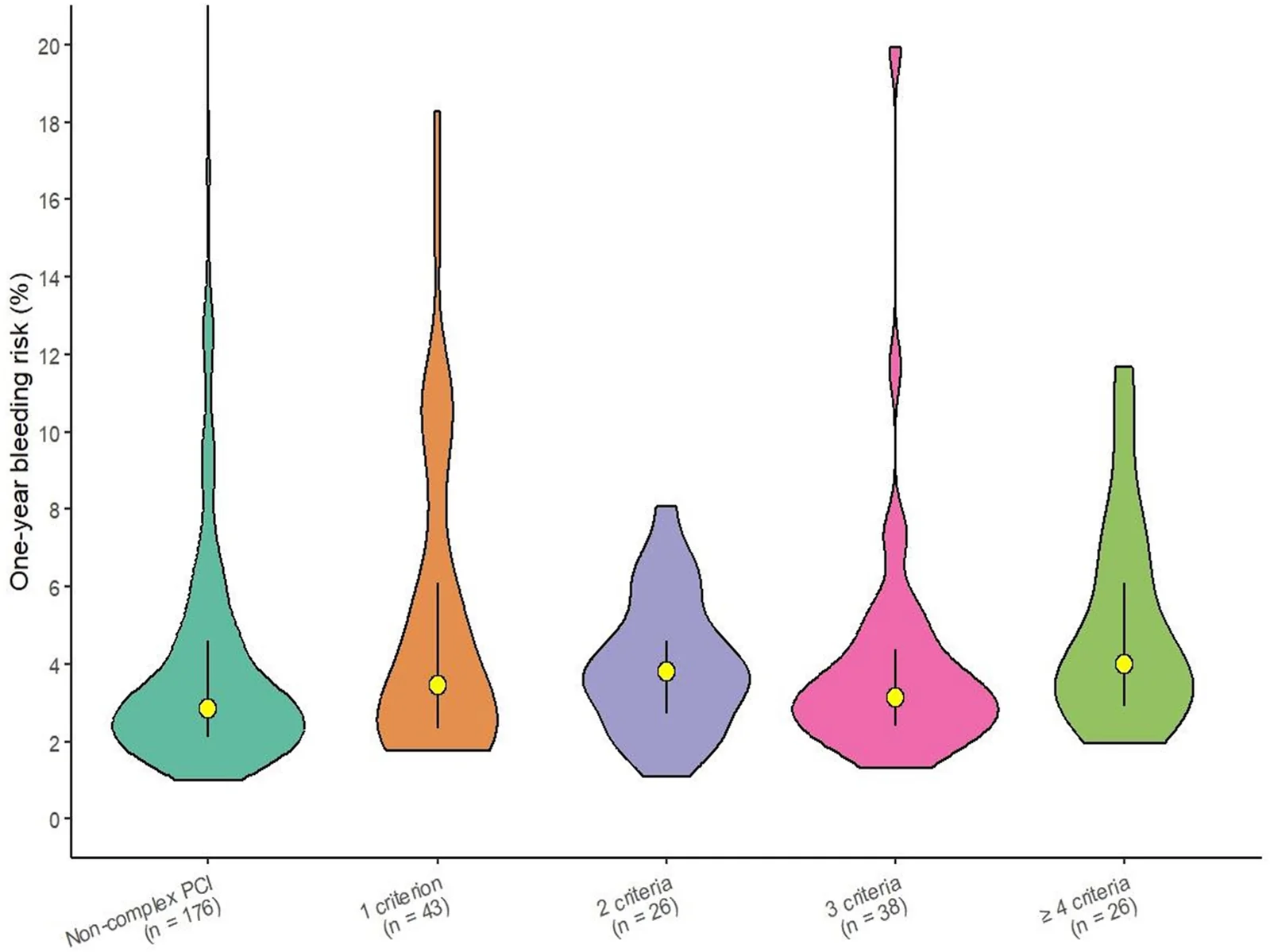
One-year bleeding risk by number of complex percutaneous coronary intervention (PCI) criteria in total study population. Violin plots illustrate the distribution density of estimated 1-year major bleeding risk across increasing levels of PCI complexity. One-year bleeding risk was calculated using the **Pre**dicting Bleeding **C**omplications **i**n Patients Undergoing **S**tent Implantation and Subs**e**quent **D**ual **A**nti **P**latelet **T**herapy-high bleeding risk (PRECISE-HBR) score. The **width** of each violin represents the relative frequency of observations at a given risk value. The **central vertical line** indicates the interquartile range (25th to 75th percentile), and the **yellow point** denotes the median risk for each group. The y-axis is truncated at 20% for visualization purposes.

**Figure 2:**
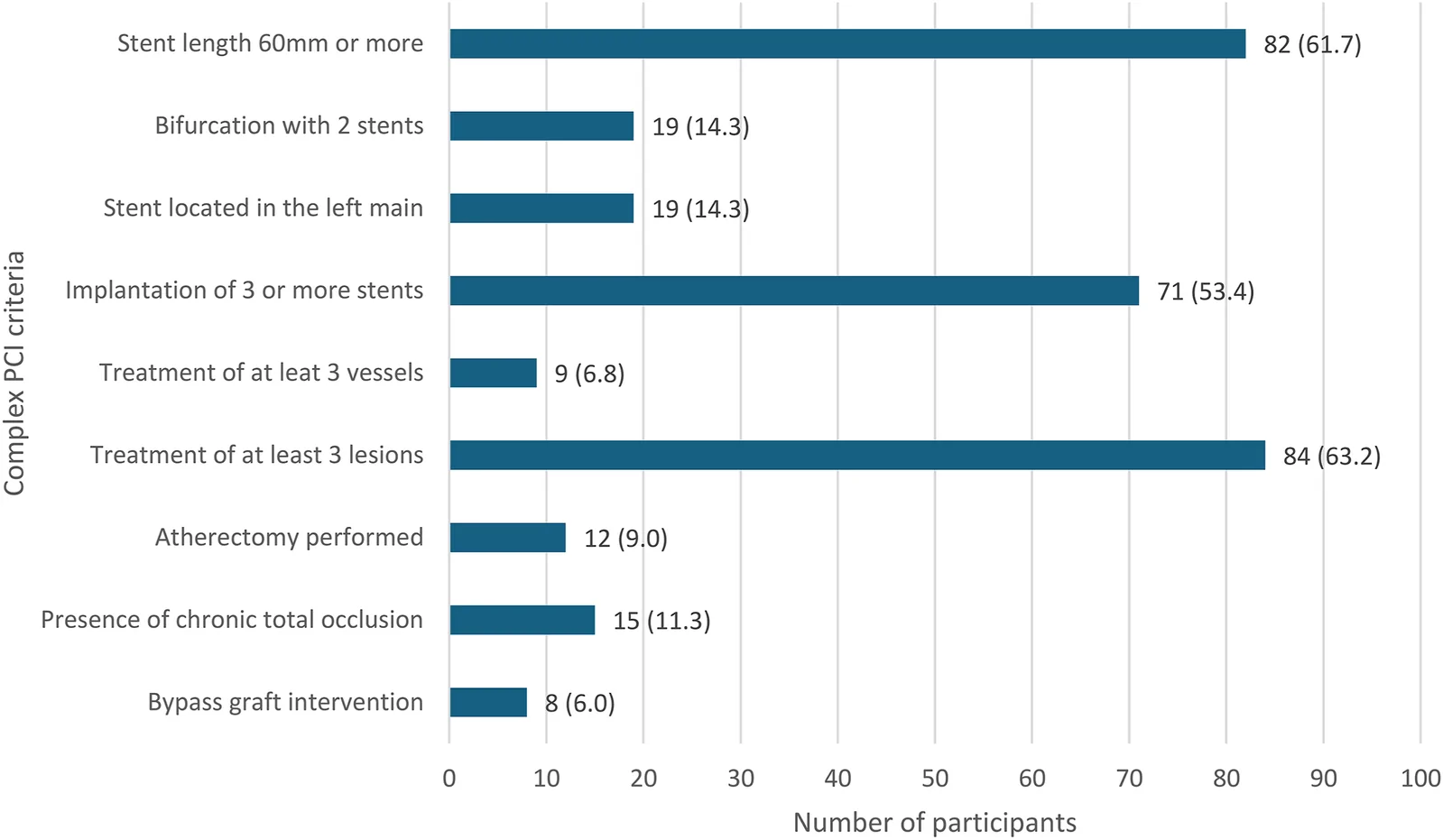
Complex percutaneous coronary intervention (PCI) criteria by number of participants. Data are expressed as n (%).

Among the 284 patients not receiving long-term anticoagulation at discharge, standard-duration DAPT with ticagrelor or prasugrel was most frequently prescribed (61.3%), followed by standard-duration DAPT with clopidogrel (34.5%). Overall, de-escalation strategies were uncommon (4.2%), including short-duration DAPT in 3.5% and reduced-intensity DAPT in 0.7% of patients. For patients prescribed short-duration DAPT, the planned single antiplatelet agent after DAPT discontinuation was most commonly a P2Y_12_ inhibitor (ticagrelor, n = 4; clopidogrel, n = 4; prasugrel, n = 1), and aspirin monotherapy was prescribed in 1 patient. Antiplatelet selection differed according to bleeding risk (Fig. 3). In non-HBR patients, standard-duration DAPT with ticagrelor or prasugrel predominated (73.2%), whereas in HBR patients, standard-duration DAPT with clopidogrel was most frequently prescribed (61.6%). However, within the HBR subgroup, the presence of complex PCI was not associated with differences in antiplatelet strategy, with similar use of standard-duration clopidogrel-based DAPT (63% vs 60%) and standard-duration potent DAPT (33% vs 35%) in complex vs non-complex PCI. A comparable lack of association between procedural complexity and antiplatelet selection was observed among non-HBR patients. Findings were similar when the vHBR definition was applied. In post hoc sensitivity analyses stratified by ACS subtype (Supplemental Figs. S3 and S4), clopidogrel use was higher in NSTEMI or unstable angina than in STEMI. Among HBR patients, standard-duration clopidogrel-based DAPT predominated in NSTEMI or unstable angina and in STEMI without complex PCI.

**Figure 3:**
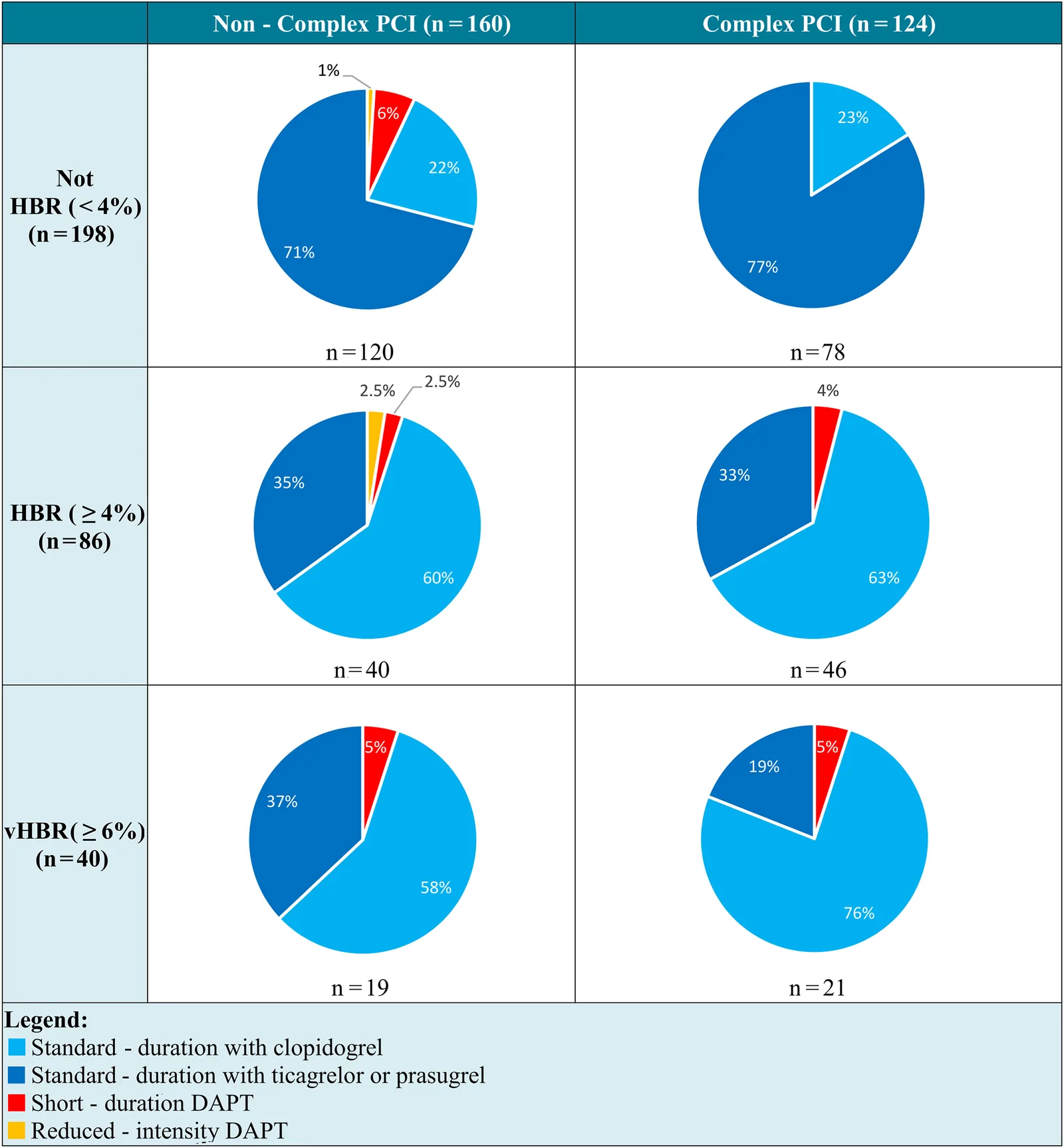
Prescribed dual antiplatelet therapy (DAPT) strategies by bleeding risk and presence of complex percutaneous coronary intervention (PCI) in participants not on long-term anticoagulation. HBR, high bleeding risk; vHBR, very high bleeding risk.

## Discussion

This study provides contemporary insight into the coexistence of bleeding and thrombotic risk after PCI for ACS. Nearly 1 in 6 patients met both HBR and complex PCI criteria, highlighting that this dual-risk phenotype is a frequent clinical scenario rather than a rare exception. Notably, guideline-endorsed de-escalation strategies were seldom adopted, even though the study period began 6 months after publication of the 2023 CCS/CAIC antiplatelet guideline update.^1^ Although bleeding risk appeared to influence antiplatelet selection (clopidogrel-based DAPT more frequently prescribed in those patients, compared to potent DAPT), standard-duration DAPT remained the predominant approach across risk strata.

These findings should be interpreted in light of contemporary real-world PCI populations and existing clinical trial data. The overall prevalence of HBR in our cohort using the PRECISE-HBR definition (35%) is consistent with large contemporary PCI registries reporting rates between approximately 27% and 44%, depending on the definition applied.^18^, ^19^, ^20^, ^21^ Notably, 9.1% of patients met the more restrictive vHBR definition according to the recently proposed PRECISE-HBR score, providing new data on this particularly vulnerable subgroup.^13^ In an exploratory sensitivity analysis using available ARC-HBR criteria, the prevalence of HBR and the overlap between HBR and complex PCI were similar, although slightly lower than with PRECISE-HBR. However, ARC-HBR-defined HBR may have been underestimated because selected ARC-HBR criteria, including intracranial bleeding risk factors, nondeferrable major surgery while receiving DAPT, and prior ischemic stroke, were not formally collected. Although PRECISE-HBR incorporates multiple clinical variables, older age, lower hemoglobin, and concomitant oral anticoagulation accounted for most HBR and vHBR classifications in our cohort, suggesting that a limited number of clinical features drive bleeding risk stratification in routine practice. In contrast, the prevalence of complex PCI in our study (43%) was higher than that reported in a large contemporary European registry, in which complex PCI—defined using largely similar criteria—was observed in 27.3% of patients.^22^ This difference may reflect our status as a tertiary referral cardiology centre, where patients with more anatomically complex or high-risk coronary disease are frequently treated. The most frequent complex features were also comparable, with ≥ 3 stents and a ≥ 60 mm stent length among the top criteria in both cohorts, whereas multivessel PCI predominated in the registry and ≥ 3 lesions treated was most frequent in our study. An important finding is that we did not observe a clear association in predicted bleeding risk across increasing levels of PCI complexity, indicating that thrombotic and bleeding risk profiles do not necessarily evolve in parallel but instead might reflect distinct clinical characteristics.

Despite updated guideline recommendations, de-escalation strategies were rarely planned in our cohort, with most patients receiving a prescription with standard-duration DAPT. Of note, even among HBR patients without complex PCI, de-escalation strategies were infrequently envisaged at discharge. This limited uptake likely reflects a combination of clinical and operational barriers. Although our study was conducted 6 to 18 months after publication of the most recent Canadian antiplatelet guidelines, some degree of clinical inertia following guideline publication may have contributed to the persistent predominance of standard-duration DAPT strategies. Moreover, complex PCI increases perceived thrombotic risk, particularly in the immediate post-procedural period when the threat of stent thrombosis is tangible, whereas major bleeding risk may be viewed as more probabilistic and temporally distant. Although subanalyses of the **Ma**nagement of High Bleeding Risk Patients Post Bioresorbable Polymer Coated **Ste**nt Implantation With an Abb**r**eviated Versus Standard **DAPT** Regimen (MASTER DAPT) trial, as well as a meta-analysis of 6 RCTs, have reported that DAPT de-escalation is associated with similar rates of ischemic events and lower risk of major bleeding, compared with standard DAPT irrespective of PCI complexity, data remain scarce in patients undergoing complex PCI.^10^^,^^11^ In such settings, clinicians may be understandably reluctant to shorten DAPT duration. In addition, trials evaluating de-escalation strategies in HBR populations were primarily designed and powered to assess bleeding outcomes rather than ischemic events, which may contribute to persistent uncertainty regarding the potential ischemic tradeoff in patients with high thrombotic risk features. When high bleeding and high thrombotic risk coexist, clinicians must choose among several antiplatelet strategies. Without clear evidence or guideline recommendations favouring one approach in this overlapping risk profile, decision-making may default to established practice patterns, including the historically dominant 12-month DAPT strategy that has remained standard since the early 2000s.^23^ Operational factors may further constrain implementation of de-escalation strategies. Both de-escalation approaches require a clearly defined follow-up plan to reassess ischemic and bleeding risk and implement therapy modification at 1-3 months, which may be difficult to coordinate when postdischarge care occurs in the community. In addition, they require explicit communication regarding the rationale, timing, and therapeutic intent of planned changes, and responsibility for these modifications may be unclear across care settings. Both approaches also require patient education and clear counselling regarding anticipated therapy adjustments, which may complicate implementation within routine discharge workflows.

At the same time, our findings suggest that bleeding risk does influence therapeutic decision-making, as HBR patients were more frequently treated with standard-duration clopidogrel-based DAPT, whereas non-HBR patients more often received standard-duration potent DAPT. In contrast, the presence of complex PCI alone did not consistently modify antiplatelet selection among HBR and non-HBR patients. An exception was observed among patients presenting with both STEMI and complex PCI, in whom standard-duration potent DAPT predominated; however, this subgroup was relatively small and these findings should be interpreted with caution. Together, these observations suggest that in patients with overlapping bleeding and thrombotic risk, clinicians rarely planned to implement formal de-escalation strategies at discharge, but appeared to account for HBR by preferentially selecting standard-duration clopidogrel-based DAPT rather than standard-duration potent DAPT therapy. This finding is consistent with other published data suggesting that when concordant, bleeding, more than ischemic risk, should inform decision-making regarding antiplatelet strategy.^10^^,^^11^^,^^16^

This study has limitations. First, the retrospective, single-centre, observational design may be affected by residual confounding due to unmeasured variables, including clinical factors such as frailty or thrombus burden at index PCI and may limit the generalizability of our findings to other practice settings. However, the available data allowed application of contemporary, guideline-aligned definitions and validated tools, such as the complex PCI criteria and the recently validated PRECISE-HBR score.^1^^,^^13^ Second, although the PRECISE-HBR score is a validated tool for bleeding risk assessment after PCI, its overall predictive performance for major bleeding remains moderate (reported area under the curve approximately 0.73), which should be considered when interpreting our findings.^13^ Third, the PRECISE-HBR score was calculated only once for each patient, even though bleeding risk is dynamic and may be better represented with serial assessments. This approach still mirrors routine practice, in which reassessing bleeding risk within a few months can be challenging. In addition, the PRECISE-HBR score has been validated using baseline patient data at the time of index PCI.^13^

A key strength of this study is its setting in a tertiary cardiology centre, with high PCI volume and dedicated expertise in antiplatelet management, where antiplatelet prescribing practices are expected to be more closely aligned with updated guideline recommendations. As such, this environment may provide early insight into real-world adoption of contemporary treatment strategies. A point that should be noted, however, is that we used prescriptions at discharge to assess the DAPT planning. Although interventional cardiologists are encouraged to provide recommendations regarding DAPT strategy at discharge, subsequent antiplatelet therapy modifications may occur during longitudinal follow-up outside our institution and therefore would not have been captured in our dataset. Therefore, our findings may underestimate the eventual use of de-escalation strategies after discharge. Another strength of this study was the use of a sampling strategy in 52 blocks of 7 days, with random patient selection within each block, which helped avoid selective inclusion by data collectors and reduced the risk of overrepresenting a practice pattern that may be unique to a small subset of clinicians.

In conclusion, in contemporary PCI practice, the coexistence of HBR and procedural complexity represents a frequent and clinically relevant phenotype. Despite updated guideline recommendations, formal de-escalation strategies were infrequently planned at patient discharge, even among patients at HBR without complex PCI. Instead, clinicians appeared to account for bleeding risk primarily through selection of standard-duration clopidogrel-based DAPT, although procedural complexity alone did not appear to meaningfully influence antiplatelet selection. These findings underscore the need for clearer evidence and practical guidance to inform antiplatelet decision-making in patients with overlapping high bleeding and thrombotic risk.

## Ethics Statement

The study protocol (#2025-3499) was approved by the Montreal Heart Institute Research Ethics Board, which waived the requirement for individual patient consent. The research reported in this paper adhered to the STROBE guidelines.

## Declaration of Generative AI and AI-Assisted Technologies

During the preparation of this work, the authors used ChatGPT 5.2 from OpenAi to improve language and readability. After using this tool/service, the senior author reviewed and edited the content as needed and takes full responsibility for the content of the published article.

## Patient Consent

The authors confirm that patient consent is not applicable to this article. This is a descriptive study using de-identified data; the institutional review board did not require consent from the patient.

## Funding Sources

E.R.C., S.C., A.G., and D.T. received a scholarship from the Ministère de la Santé et des Services Sociaux du Québec. J.S. received a research initiation scholarship from the 10.13039/501100024252Faculty of Pharmacy at the Université de Montréal. M.L. is a Canada Research Chair in Platelets As Biomarkers and Vectors (950-232706). The Faculty of Pharmacy, Université de Montréal, also provided support for publication fees related to this article.

## Disclosures

M.L. was a primary member of the writing group for the Canadian Cardiovascular Society/Canadian Association of Interventional Cardiology 2023 Focused Update of the Guidelines for the Use of Antiplatelet Therapy. The other authors have no conflicts of interest to disclose.

## Footnotes

To access the supplementary material accompanying this article, visit *CJC Open* at https://www.cjcopen.ca/ and at https://doi.org/10.1016/j.cjco.2026.06.009
