# Supplementary material for: Antiplatelet Therapy Strategies in Patients with High Bleeding Risk Treated with Complex Percutaneous Coronary Intervention: A Descriptive Study
Source: CJC Open. 2026 Jul 1;8(10):1389–98. doi: 10.1016/j.cjco.2026.06.009 (PMC13654379; doi:10.1016/j.cjco.2026.06.009)
Supplement: Supplementary file 1 [file mmc1.docx]

# **Supplemental Figure S1** Flow of participants through the study

**Identification of patients**

Patients excluded:

Reason for PCI other than ACS (n = 15)

Planned cardiac surgery during index hospitalization (n = 10)

History of stent thrombosis (n = 10)

Did not receive stent (n = 2)

Died (n = 1)

Included in another study (n = 1)

Thrombophilia (n = 1)

Patients assessed for eligibility

(n = 349)

**Screening**

Patients included in study

(n = 309)

**Included**

**Abbreviations:** ACS = acute coronary syndrome; PCI = percutaneous coronary intervention

# **Supplemental Table S1**. Proportional Overlap Between High Bleeding Risk (PRECISE-HBR) and Complex PCI

|  | Proportion of patients by clinical risk features |
| --- | --- |
| Total study population (n=309) | |
| Patients with HBR only | 17.5 (13.3 - 21.7) |
| Patients with Complex PCI only | 25.5 (20.6 - 30.4) |
| Patients with both HBR and Complex PCI | 17.5 (13.3 - 21.7) |
| Patients with neither HBR nor Complex PCI | 39.5 (34.0 - 45.0) |
| Participants not on long-term anticoagulation (n=284) | |
| Patients with HBR only | 14.1 (10.1 - 18.1) |
| Patients with Complex PCI only | 27.5 (22.3 - 32.7) |
| Patients with both HBR and Complex PCI | 16.2 (11.9 - 20.5) |
| Patients with neither HBR nor Complex PCI | 42.2 (36.6 - 48.0) |
| Total study population (n=309) | |
| Patients with vHBR only | 9.4 (6.1 - 12.6) |
| Patients with Complex PCI but not vHBR | 34.0 (28.7 – 39.3) |
| Patients with both vHBR and Complex PCI | 9.1 (5.9 – 12.3) |
| Patients with neither vHBR nor Complex PCI | 47.5 (42.0 – 53.1) |

**Legend**: Data are expressed in % (95% CI)

**Abbreviations:** HBR = high bleeding risk (≥4%); PCI = percutaneous coronary intervention; vHBR = very high bleeding risk (≥6%)

# **Supplemental Figure S2**. Proportional Overlap Between High Bleeding Risk (ARC-HBR) and Complex PCI

a. Total study population (n=309)

Complex PCI only

**29.1%**
n=90

Both

**13.9%**
n=43

HBR
only **14.9%**
n=46

Neither risk **42.1%**
n=130

b. Participants not on long-term anticoagulation (n=284)

Complex PCI only

**37.1%**
n=90

Both

**12.0%**
n=34

HBR
only **10.6%**
n=30

Neither risk **45.8%**
n=130

**Legend:** Two Venn diagrams illustrate the overlap between HBR and complex PCI in the study population. Circle areas are proportional to the number of patients in each category. **Panel A** depicts the overall cohort using an HBR definition of ARC-HBR. **Panel B** shows the same analysis restricted to patients not receiving long-term oral anticoagulation.

**Abbreviations:** ARC-HBR **=** Academic Research Consortium for High Bleeding Risk; HBR = high bleeding risk; PCI = percutaneous coronary intervention

# **Supplemental Table S2**. Proportional Overlap Between High Bleeding Risk (ARC-HBR) and Complex PCI

|  | Proportion of patients by clinical risk features |
| --- | --- |
| Total study population (n=309) | |
| Patients with HBR only | 14.9 (10.9 - 18.9) |
| Patients with Complex PCI only | 29.1 (24.1 - 34.2) |
| Patients with both HBR and Complex PCI | 13.9 (10.1 - 17.8) |
| Patients with neither HBR nor Complex PCI | 42.1 (36.6 - 47.6) |
| Participants not on long-term anticoagulation (n=284) | |
| Patients with HBR only | 10.6 (7.0 - 14.1) |
| Patients with Complex PCI only | 31.7 (26.3 - 37.1) |
| Patients with both HBR and Complex PCI | 12.0 (8.2 - 15.7) |
| Patients with neither HBR nor Complex PCI | 45.8 (40.0 - 51.6) |

**Legend**: Data are expressed in % (95% CI)

**Abbreviations:** ARC-HBR **=** Academic Research Consortium for High Bleeding Risk; HBR = high bleeding risk; PCI = percutaneous coronary intervention

# **Supplemental Table S3**. One-year major bleeding risk by number of complex PCI criteria in total study population

|  | Minimum value | 25^th^ percentile | Median | 75th percentile | Maximum value |
| --- | --- | --- | --- | --- | --- |
| Non-complex PCI (n=176) | 1.0% | 2.1% | 2.9% | 4.6% | 31.8% |
| 1 criterion (n=43) | 1.8% | 2.4% | 3.5% | 6.1% | 18.3 % |
| 2 criteria (n=26) | 1.1% | 2.7% | 3.8% | 4.6% | 8.1 % |
| 3 criteria (n=38) | 1.3% | 2.4% | 3.1% | 4.4% | 19.9 % |
| ≥ 4 criteria  (n=26) | 1.9% | 2.9% | 4.0% | 6.1% | 11.7 % |

**Legend**: Values indicate the estimated one-year risk of major bleeding after PCI, calculated using the PRECISE-HBR score.
**Abbreviation**: PCI = percutaneous coronary intervention.

# **Supplemental Figure S3**. Prescribed DAPT strategies by bleeding risk and presence of complex PCI in participants with STEMI not on long term anticoagulation

|  | **Non-complex PCI (n=76)** | **Complex PCI (n=69)** |
| --- | --- | --- |
| **Not HBR (<4%)**  **(n=99)** | n=57 | n=42 |
| **HBR (≥4%)**  **(n=32)** | n=12 | n=20 |
| **vHBR (≥6%)**  **(n=14)** | n=7 | n=7 |
| **Legend:**  Standard-duration with clopidogrel  Standard-duration with ticagrelor or prasugrel  Short-duration DAPT Reduced-intensity DAPT | | |

|  | Ventes |
| --- | --- |
| 1er trim. | 0% |
| 2e trim. | 5% |
| 3e trim. | 40% |
| 4e trim. | 55% |

|  | Ventes |
| --- | --- |
| 3e trim. | 0.58 |
| 4e trim. | 0.42 |

|  | Ventes |
| --- | --- |
| 1er trim. | 2% |
| 2e trim. | 2% |
| 3e trim. | 7% |
| 4e trim. | 89% |

|  | Ventes |
| --- | --- |
| 1er trim. | 0% |
| 2e trim. | 0% |
| 3e trim. | 12% |
| 4e trim. | 88% |

|  | Ventes |
| --- | --- |
| 1er trim. | 0% |
| 2e trim. | 0% |
| 3e trim. | 71% |
| 4e trim. | 29% |

|  | Ventes |
| --- | --- |
| 2e trim. | 0% |
| 3e trim. | 71% |
| 4e trim. | 29% |

**Abbreviations:** DAPT = dual antiplatelet therapy; HBR = high bleeding risk; PCI = percutaneous coronary intervention; STEMI = ST-elevation myocardial infarction; vHBR = very high bleeding risk

# **Supplemental Figure S4**. Prescribed DAPT strategies by bleeding risk and presence of complex PCI in participants with NSTEMI or unstable angina not on long term anticoagulation

|  | **Non-complex PCI (n=103)** | **Complex PCI (n=76)** |
| --- | --- | --- |
| **Not HBR (<4%)**  **(n=99)** | n=63 | n=36 |
| **HBR (≥4%)**  **(n=54)** | n=28 | n=26 |
| **vHBR (≥6%)**  **(n=26)** | n=12 | n=14 |
| **Legend:**  Standard-duration with clopidogrel  Standard-duration with ticagrelor or prasugrel  Short-duration DAPT Reduced-intensity DAPT | | |

|  | Ventes |
| --- | --- |
| 1er trim. | 0% |
| 2e trim. | 4% |
| 3e trim. | 81% |
| 4e trim. | 15% |

|  | Ventes |
| --- | --- |
| 1er trim. | 3% |
| 2e trim. | 4% |
| 3e trim. | 61% |
| 4e trim. | 32% |

|  | Ventes |
| --- | --- |
| 1er trim. | 0% |
| 2e trim. | 10% |
| 3e trim. | 36% |
| 4e trim. | 54% |

|  | Ventes |
| --- | --- |
| 1er trim. | 0% |
| 2e trim. | 0% |
| 3e trim. | 36% |
| 4e trim. | 64% |

|  | Ventes |
| --- | --- |
| 1er trim. | 0% |
| 2e trim. | 8% |
| 3e trim. | 50% |
| 4e trim. | 42% |

|  | Ventes |
| --- | --- |
| 2e trim. | 7% |
| 3e trim. | 79% |
| 4e trim. | 14% |

**Abbreviations**: DAPT = dual antiplatelet therapy; HBR = high bleeding risk; NSTEMI = Non-ST-segment elevation myocardial infarction; PCI = percutaneous coronary intervention; vHBR = very high bleeding risk
